# Evaluating efforts to diversify the biomedical workforce: the role and function of the Coordination and Evaluation Center of the Diversity Program Consortium

**DOI:** 10.1186/s12919-017-0087-4

**Published:** 2017-12-04

**Authors:** Heather E. McCreath, Keith C. Norris, Nancy E. Calderόn, Dawn L. Purnell, Nicole M. G. Maccalla, Teresa E. Seeman

**Affiliations:** 1 Department of Medicine, David Geffen School of Medicine, Division of Geriatrics, University of California, Los Angeles, CA 90095 USA; 2 Department of Medicine, David Geffen School of Medicine, Division of General and Internal Medicine, University of California, Los Angeles, CA 90095 USA; 3 Department of Education, Graduate School of Education and Information Studies, University of California, Los Angeles, CA 90095 USA

## Abstract

**Background:**

The National Institutes of Health (NIH)-funded Diversity Program Consortium (DPC) includes a Coordination and Evaluation Center (CEC) to conduct a longitudinal evaluation of the two signature, national NIH initiatives - the Building Infrastructure Leading to Diversity (BUILD) and the National Research Mentoring Network (NRMN) programs - designed to promote diversity in the NIH-funded biomedical, behavioral, clinical, and social sciences research workforce. Evaluation is central to understanding the impact of the consortium activities. This article reviews the role and function of the CEC and the collaborative processes and achievements critical to establishing empirical evidence regarding the efficacy of federally-funded, quasi-experimental interventions across multiple sites. The integrated DPC evaluation is particularly significant because it is a collaboratively developed Consortium Wide Evaluation Plan and the first hypothesis-driven, large-scale systemic national longitudinal evaluation of training programs in the history of NIH/National Institute of General Medical Sciences.

**Key highlights:**

To guide the longitudinal evaluation, the CEC-led literature review defined key indicators at critical training and career transition points – or Hallmarks of Success. The multidimensional, comprehensive evaluation of the impact of the DPC framed by these Hallmarks is described. This evaluation uses both established and newly developed common measures across sites, and rigorous quasi-experimental designs within novel multi-methods (qualitative and quantitative). The CEC also promotes shared learning among Consortium partners through working groups and provides technical assistance to support high-quality process and outcome evaluation internally of each program. Finally, the CEC is responsible for developing high-impact dissemination channels for best practices to inform peer institutions, NIH, and other key national and international stakeholders.

**Implications:**

A strong longitudinal evaluation across programs allows the summative assessment of outcomes, an understanding of factors common to interventions that do and do not lead to success, and elucidates the processes developed for data collection and management. This will provide a framework for the assessment of other training programs and have national implications in transforming biomedical research training.

## Background

The Coordination and Evaluation Center (CEC) serves as a critical link in the activity and vision of the Diversity Program Consortium (DPC) effort. As an entity separate from the funding agency – the National Institutes of Health (NIH) - and the programs implementing targeted activities – the Building Infrastructure Leading to Diversity (BUILD) and National Research Mentoring Network (NRMN) initiatives - the CEC has distinct and unique functions. The main role of the CEC is to conduct an evaluation of the DPC initiatives across programs, with a focus on long-term outcome assessment. In contrast, BUILD and NRMN were designed to develop and test novel and innovative programs to increase the number of well-trained NIH-funded investigators from underrepresented groups. The resulting scope of the DPC is broad, spanning multiple career stages that represent critical years of investment in training and education for biomedical scientists. Shared identification of existing measures and development of new measures leverages the power of the Consortium and contributes to local program evaluation and the scholarship on training for biomedical research careers. To frame the expansive evaluation of the DPC, the CEC worked with a committee of representatives from each initiative awardee and the NIH program office to identify the important indicators of transition through career stages that are being addressed across the consortium, or DPC Hallmarks of Success. Using these Hallmarks, the CEC has designed a multidimensional, comprehensive assessment of the impact of the DPC utilizing common measures within novel multi- methods (qualitative and quantitative).

Connecting the BUILD and NRMN initiatives in a comprehensive assessment approach enables the NIH to discern and promote dissemination of generalizable knowledge, including best practices that optimize institutional commitment to diversity, faculty and underrepresented student’s career success in biomedical science. Critical to accomplishing these goals are partnerships with a variety of stakeholders: students and faculty at a wide variety of institutions, fellow grantees and investigators, NIH leadership and staff, institutional leaders at DPC-funded institutions, professional societies, and biomedical training programs with related goals and objectives. The CEC is charged with coordinating activities and communication across the Consortium. Dissemination of tools, approaches, and findings will extend the investment in the Consortium and the CEC serves a central role in such activities.

Thus, the integrated DPC evaluation is particularly significant because it is one of the few national, longitudinal evaluations of training programs for the National Institute of General Medical Sciences. The Consortium-Wide Evaluation Plan is the largest longitudinal, hypothesis-driven diversity pipeline evaluation ever funded by the NIH. The findings of the initiative, including the processes for data collection and management, will serve as a national model for biomedical training and assessment.

## Hallmarks of success

**Fig. 1:**
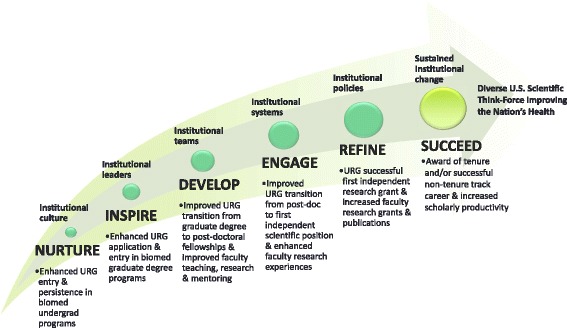
Diversity Program Consortium Arc of Success for biomedical research

A key challenge to the evaluation of the DPC initiatives is that the trajectory for undergraduate students (~age 20) to NIH R01-awarded researcher (~age 42) is complex and involves a longer period than the time frame of the federal funding provided. There are many transition points along that trajectory where students may be more vulnerable. For instance, underrepresented minorities intending to major in science, technology, engineering and mathematics (STEM) are twice as likely as white students to switch to a non-STEM field before graduation from college [1]. Thus, the CEC developed an “Arc of Success” to delineate stages along this trajectory (Figure 1). Participants in DPC initiatives reflect individuals from across this arc, with some entering as undergraduate STEM students while others may become involved as they transition from graduate degree to post-doctoral positions or first faculty positions. Still others may be in faculty positions but not yet have had an NIH grant funded. Thus, a range of longitudinal and cross-sectional data collection modalities and strategies need to be utilized for assessing the variety of experiences and accomplishments that predict a high likelihood of future success in biomedical research, academia, and NIH funding along the career trajectory.

Accordingly, the DPC developed a set of consortium-wide Hallmarks, or potential predictors of successful transitions across the Arc of Success – essentially, key career transition points. Some Hallmarks are both outcomes at early stages and predictors for later stages of the Arc of Success. By defining Hallmarks, the DPC can measure and track the path for individuals in a way that can be summarized and analyzed for patterns that promote and inhibit progress [2].

Traditionally, it has been difficult to assess relatively matched cohorts of trainees using such hallmarks. One barrier has been the paucity of consistent and reliable evaluative data [3]. Often, programs are not provided with direction on what type of evaluation data to collect so that common measures can be employed across programs targeting different points along the career trajectory [4]. Participating stakeholders may not understand the importance of providing all the information requested or there may not have been clear communication about a data reporting schedule. In many cases, the funding for collection of evaluation data may be limited or not included in the project budget.

Another barrier has been bias in student participation and program reporting of interventions. For instance, programs may work to maximize limited resources by narrowing the selection criteria for participating students. While quasi-experimental approaches can be used to define appropriate comparisons, funding agencies may not emphasize the importance of such designs or may not have adequate resources to support such designs [4].

There is also lack of agreement on how confidential data that could be used for such evaluations should be disaggregated across disciplines, racial groups, and gender [4]. It is important to maintain the confidentiality of program participants. This may become difficult if data are examined by multiple strata, perhaps resulting in making it possible to identify the five women biochemists who are participating in a grant-writing workshop. Data structures and reporting templates can be designed to minimize the risks of identification, but such tools need to be explicitly designed and incorporated into the evaluation.

Finally, collecting longitudinal data to generate and test causal models linked to successful participation in STEM for diverse student populations has been challenging due to evolving appreciation for privacy and confidentiality issues [4]. Over the last several years, programs have worked with their Institutional Review Boards to determine when it is necessary to incorporate language about consent to contact participants for follow-up. Funding for longitudinal contacts can also be difficult to obtain, as downstream effects may need to be measured several years after the original program.

The CEC worked with the DPC Executive Steering Committee (composed of PIs and investigators from the funded initiatives and NIH) in a collaborative effort to establish the consortium-wide Hallmarks of Success. First, the CEC conducted an extensive literature search including several NIH reports recommending goals of diversity training programs [5].

**Fig. 2:**
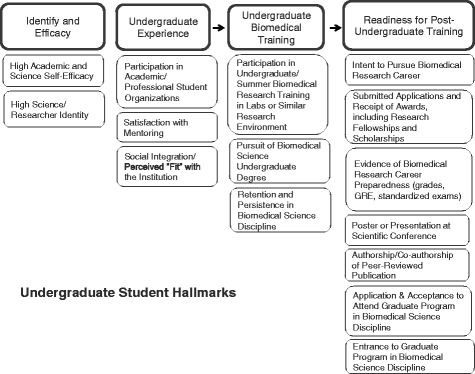
Diversity Program Consortium Hallmarks of Success for undergraduate students and mentees

**Fig. 3:**
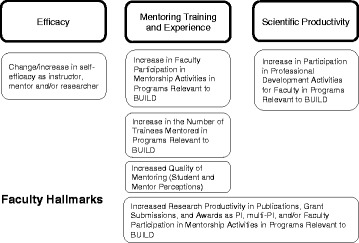
Diversity Program Consortium Hallmarks of Success for faculty and mentors

**Fig. 4:**
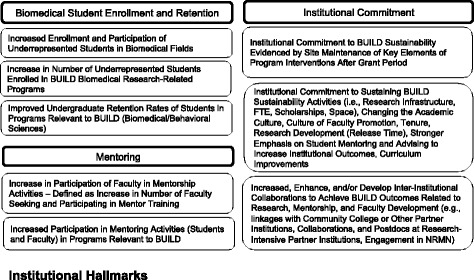
Diversity Program Consortium Hallmarks of Success for institutions

From the literature review and NIH model, the DPC Executive Steering Committee completed an iterative review process to establish the Hallmarks. The guiding principle was that the Hallmarks needed to be relevant to a majority of the DPC programs and measurable in a consistent manner across DPC program sites for a broad array of activities and institutional interventions. The DPC Hallmarks of Success include traditional, non-traditional, and potentially novel hallmarks based on prior experience and planned activities and interventions of the DPC program awardees. Figures 2, 3 and 4 present a summary of the Hallmarks that received collective approval across the Consortium. For organizational purposes, the Hallmarks of Success were divided into three domains: Student/Mentee, Faculty/Mentor, and Institutional. Within each domain, Hallmarks are clustered conceptually. Hallmarks relevant for individual mentees may be classified in student or faculty domains, depending on the career level of the individual. Student and faculty Hallmarks can also be viewed as defining the beginning, intermediate, and advanced career levels and may often overlap.

The DPC Hallmarks of Success are designed to be comprehensive, encompassing various levels of student and faculty activities – each known or hypothesized to have a positive influence on the probability that the individual will pursue a successful career in biomedical research. Each of these Hallmarks is not essential. Some researchers, for example, may seek graduate training in health sciences after completing undergraduate degrees in other fields. Others may experience rewarding research activity without attending a summer enrichment program. The Hallmarks can be viewed as a set of key indicators of the likelihood of success in pursuing a career in biomedical research. The cumulative effect of achieving Hallmarks is one of the scientific questions the DPC will provide data to address. Generally, it is hypothesized that the more of these Hallmarks achieved, the higher the likelihood of a successful biomedical research career.

These Hallmarks provide a framework within which to assess factors contributing to different career paths. In the case of the BUILD and NRMN initiatives, the Hallmarks can be monitored longitudinally and compared within and across institutions to understand program impact and collective progress in unique institutional settings. Such longitudinal study may suggest that certain Hallmarks are more critical experiences or accomplishments for certain groups of individuals. For instance, participating in research as an undergraduate appears to be more critical as an experience in predicting pursuit of biomedical graduate school training for first-generation college students compared with students whose family members received college degrees [6]. Thus, the robust analysis of Hallmarks will allow tailored recommendations to advance national strategies to increase diversity in high quality students committed to careers in the biomedical sciences. A brief overview of the Hallmarks is described below.

*Student/mentee Hallmarks* are a mix of psychosocial factors and educational and professional accomplishments and experiences (Figure 2). Some individual factors are specific to scientific and biomedical careers, such as development of science identity, scientific self-efficacy, and aspirations for scientific research [7, 8]. Other factors are more general, such as academic self-efficacy, social integration and sense of belonging in the university setting [1, 9–11]. Increases for undergraduate students on these factors are considered hallmarks that represent early predictors of successful longer term outcomes such as completing graduate work in biomedical research and becoming an independent scientist (either as research faculty or a senior scientist in the private sector).

Accomplishments and experiences noted as student/mentee Hallmarks are focused on retention and persistence in biomedical research training [6, 8, 10, 12]. Key Hallmarks specifically for undergraduates are participation in biomedical research activities that include presentations at scientific conferences [5, 13, 14] and matriculation to graduate biomedical programs [5]. Matriculation is viewed as both a successful outcome of the undergraduate experience and an important predictor of future career success in the biomedical sciences.

*Faculty/mentor-level Hallmarks* include program-relevant activities and behaviors among faculty, including increased participation in professional development activities for improving teaching and in mentorship activities and training, as well as improvement in the quality of mentoring based on identified behaviors and attitudes [15–17] (Figure 3). Hallmarks that are accomplishments are increased research productivity in publications, grant submissions and awards. Finally, psychosocial Hallmarks for faculty and mentors include change or increase in self-efficacy as instructor, mentor or researcher [17–19].

*Institutional-level Hallmarks* include the availability of resources and opportunities for students and faculty that are associated with their development (Figure 4). For example, the availability of pre-professional and departmental clubs for students, supportive and collaborative science environments, and viable research opportunities for financial assistance have been shown to enhance student success [9, 12, 20]. Compared to other institutions, successful institutions can document increased inter-institutional collaborations, improved undergraduate retention rates of students in BUILD relevant programs, increased participation in program-relevant mentoring activities (students and faculty) [21], institutional commitment to sustaining initiatives evidenced by maintenance of key elements of program interventions after grant period, the availability of undergraduate research [22], and an increase in the number of faculty seeking and participating in mentor training.

In sum, the DPC Hallmarks are a comprehensive set of training and career exposures and outcomes which evidence indicate are associated with biomedical research career success. These hallmarks are being assessed longitudinally in students and faculty and will be used to evaluate the interventions implemented by the BUILD and NRMN programs.

## Consortium-wide evaluation plan (CWEP)

### Development of the consortium-wide evaluation plan

Establishment of common indicators and outcomes of success as outlined by the Hallmarks form the base of a rigorous and holistic view of the impact of the BUILD and NRMN programs on students, faculty, and institutions in a single evaluation design. Common measures can then be identified for use across both programs (e.g., measures of science identity, researcher self-efficacy). Further, the evaluation must also be sophisticated enough to measure interventions at varying levels and contextually sensitive to allow its use across multiple sites simultaneously. It will also need to support longitudinal and cross-sectional analyses of relatively matched cohorts of trainees and young faculty to more rigorously define hallmarks linked to NIH funding success.

**Fig. 5:**
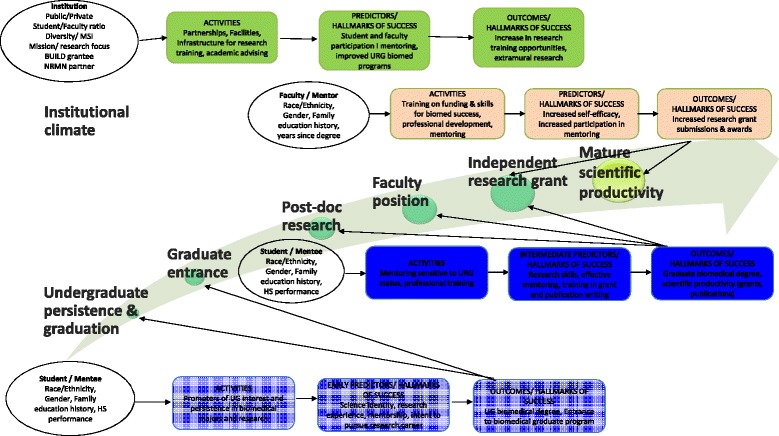
Consortium-wide conceptual model for guiding evaluation of student, mentee, faculty, mentor, and institutional outcomes

The CEC planned a comprehensive process and impact evaluation of the Diversity Program Consortium, which was refined as the Hallmarks of Success were developed and the details of the DPC initiatives and programs within each site were solidified. Fig. 5 shows conceptually how the evaluation plan builds from the Arc of Success. Activities are developed as part of the initiatives. For individuals, pre-existing characteristics may promote or hinder involvement in current and future activities. As activities accumulate, they lead to intermediate steps or hallmarks. Over time, intermediate Hallmarks increase the probability of successful long-term outcomes that are key elements on the Arc of Success. On Fig. 5, this progression is represented for three levels of individuals’ career trajectories: 1) undergraduate students, 2) graduate students and early-career faculty, and 3) senior faculty. For instance, undergraduates bring their family history and high school performance to activities that promote science identity and intent to pursue a career in biomedical research. These intermediate outcomes lead to entrance to biomedical graduate programs, the second key element on the Arc. In addition to modeling progression for individuals, institutions and organizations are also modeled this way, as institutions can systemically contribute to career progression and key Arc elements.

This conceptual model served as the underpinning to the development of Consortium-Wide Evaluation Plan (CWEP). Within this model, the resulting evaluation tracks BUILD/NRMN development at student/mentee, faculty/mentor, and institutional levels over time across career stages (e.g., from undergraduate through graduate student status and across faculty career transitions). This model also supports evaluations of short-term outcomes, such as increased science identity among students, as well as the outcomes more distal to intervention activities such as biomedical degree completion and securing independent NIH funding. It is important to note that this framework represents constructs assembled from the variety of programs proposed through the BUILD and NRMN initiatives. While the CWEP encompasses assessment of all major Hallmarks, the evaluations of the individual BUILD and NRMN programs are tailored to the specific program interventions and do not all encompass the entire set of Hallmarks or the Arc of Success. While the CWEP contains specific plans to evaluate outcomes of the BUILD and NRMN initiatives with their respective foci on undergraduates (BUILD) versus later post-doctoral scholars and junior faculty career stages (NRMN), the use of common approaches and measures allows for an overarching assessment of the full consortium efforts and impact across the Arc of Success.

### Operationalization of CWEP

A multi-methods approach is needed to collect the range of data needed to implement the CWEP evaluation. Methods include the use of common measures across all programs incorporated into longitudinal surveys for BUILD students and faculty as well as NRMN mentees and mentors. In-depth, qualitative interviews with program and partner staff, administrators, faculty/mentors and students/mentees involved in BUILD or NRMN permits an understanding of the unique contexts at each program. Observational data and institutional and program data from the various BUILD institutions and from the BUILD and NRMN programs allows for rich comparisons. For the BUILD initiative, internal institutional comparison groups and external (non-BUILD) comparison groups were created, for example using survey data gathered from program participants, non-participant students and faculty within each BUILD campus, and students at other similar types of institutions without the BUILD program. For the NRMN initiative, internal comparison group survey data is gathered from participants attending various NRMN training components. Those participating in one set of activities serve as the comparison group for those participating in another set of activities. A second comparison group is composed of those that initiated registration with NRMN but did not participate in any mentoring training activities. These comparisons permit multiple ways of assessing program impact.

Data sources were developed to support evaluation logic models for BUILD and NRMN, building from the conceptual model (see other chapters in this issue for specific evaluation details [23, 24]). CEC investigators collaborated with BUILD and NRMN sites in selecting existing items/tools to measure psychosocial measures, demographic and pre-existing characteristics, and key career outcomes defined by Hallmarks. The CEC developed a proposed plan for collection of these common measures, which was then reviewed and approved by all DPC members.

*Psychosocial predictors* include factors such as science identity, science and academic self-efficacy, satisfaction with mentorship and intent to pursue a biomedical career (see www.diversityprogramconsortium.org/datasources). Data on science identity, for example, will be collected for undergraduate (UG) students based on scales developed by a variety of investigators [6, 8, 13]. Items are included in a series of standard national surveys conducted by the Higher Education Research Institute (see below) and Consortium-administered surveys to collect this information annually begin when the student enters that institution. Parallel items are administered to faculty as well as for NRMN participants, including undergraduate students, graduate students, post-docs and faculty.

*Short and medium-term outcomes* include, for BUILD and NRMN students/mentees as well as faculty/mentors, engagement in research, presentations at scientific conferences, and authorships, as well as participation in student/professional organizations for students/mentees. For BUILD faculty, additional shorter-term outputs and outcomes include participation in BUILD faculty development activities, in BUILD mentorship programs and increases in the number of trainees mentored in BUILD programs. For NRMN, outcomes for mentees also include improved grant and career skills as well as better science networks, while those for mentors/coaches include improved quality of culturally-responsive mentoring, satisfaction with mentoring, and improved coaching skills.

*Longer-term outcomes* include some specific to undergraduates – obtaining undergraduate biomedical degree, application to graduate school and attending graduate school – as well as those for advanced trainees and faculty, who may be participating as undergraduate instructors, as mentors at all levels, or as mentees themselves – increased numbers of grant submissions and grant awards, increased publications, improved quality of mentoring (including more culturally-responsive mentoring), and increased number of students and junior faculty mentored/coached in culturally-responsive ways.

The BUILD and NRMN initiatives include important recognition of the contexts in which biomedical training and career activities occur. Therefore, data are being collected on *institutional characteristics* (for BUILD programs) as well as BUILD and NRMN program-specific information, including numbers of students from under-represented groups enrolled and retained in biomedical disciplines, numbers of students and faculty participation in mentoring, increased opportunities for student and faculty research training and greater inter- and intra-institutional collaborations. These data are collected from institutions participating in BUILD programs (e.g., descriptive statistics regarding student and faculty populations, curricula, NIH funding) as well as program surveys and institutional case studies for BUILD and interviews of NRMN network participants and observations of NRMN programs and meetings.

A series of more *in-depth qualitative interviews* and observational data collection is being conducted with subsets of BUILD and NRMN program and partner representatives as well as faculty and student program participants in order to develop a more comprehensive understanding and produce a holistic, in-depth description of programs in their effort to advance underrepresented groups in biomedical research training and success. In particular, it is important to obtain a better understanding how BUILD and NRMN sites are implementing institutional, faculty/mentor, and student/mentee interventions, as well as the development of partnerships that support and enhance the work. Special attention is given to successes and challenges, and *how* programs are able to enhance the capacity to attract, serve, and promote the success of under-represented groups in biomedical research. Overall institutionalization of programming and sustainability is also investigated. In this way, qualitative data collection serves as an opportunity to document the degree to which programs are fulfilling project objectives and initiative goals.

*Participants* in the evaluation of consortium activities include students, faculty that have participated in BUILD activities, mentees and mentors that have participated in NRMN activities, students and faculty who have not participated in any activities (both at BUILD and non-BUILD institutions), and those initially interested in NRMN who have not participated in activities. BUILD faculty participants are all from biomedical disciplines whereas BUILD student participants are from a mix of biomedical and non-biomedical majors. A full description of the sampling approaches used in the BUILD and NRMN evaluations can be found elsewhere in this volume [23, 24].

The range of interventions proposed by the BUILD and NRMN programs is extensive. As suggested in the federal funding announcement and request for proposal applications, however, there are common elements across all. To identify these common elements, the CEC worked with the other DPC partners to develop a set of *intervention categories* (see Table 1). Categories are relevant to the target group of interested (e.g., undergraduate students). Specific activities within each program were then classified into these categories. Participants are tracked regarding their involvement in each of these intervention categories. This will enable insight into how program interventions promote progress through various Hallmarks.

**Table 1:** Program activity categories by target group

Target Group	Activity Categories
BUILD Students	• Mentoring • Research Training and Support • Enrollment in Novel Curricula • Financial Support • Diversity Training • Academic Advising and Support • Career Advancement and Development
BUILD Faculty	• Mentor Training • Mentoring (as Mentee or Mentor) • Research Training and Support • Developing and Delivering Novel Curricula • Financial Support • Diversity Training
BUILD Institutions	Support for all BUILD Student and Faculty Activities, plus • Building and Development of Facilities • Collaboration and Communication • Procedures for Administration of Programs • Student Recruitment and Retention Strategies
NRMN	• Match / Link Mentors and Mentees • Mentor/Coach/ Mentee Training • Referral to Resources • Promotion of Mentoring Value

### Implementation

The consortium-wide evaluation plan (CWEP) calls for comparable sampling and timing of data collection across the Consortium. In contrast to a traditional multi-site study, the CEC worked with each of the 10 BUILD programs to accommodate local institutional contexts, including the design and implementation of the BUILD intervention, the academic year schedule and factors relating to human subjects review (e.g., Institutional Review Boards) and institutional data access. Implementation issues were relevant for NRMN as well. While institutional factors were not as prevalent, the NRMN initiative encountered participants in very different ways – in person versus online, asynchronous versus synchronous, short-term versus long-tem – and adjustments to the sampling and data collection for the CWEP were required.

To accommodate the need for such site-specific collaboration, the CEC developed implementation teams for each BUILD site and for NRMN. Each team consists of members from one consortium awardee as well as investigators and staff from each CEC core operational group (data collection, evaluation, and administrative), with one CEC junior investigator serving as lead. Teams provide regular reports to their respective groups, allowing discussion about specific questions and concerns regarding CWEP implementation. In particular, these meetings provide regular ways to ensure the data needed to address the CWEP are continuing to be collected. Occasionally, an issue is critical enough to involve the institution’s administrators. The BUILD program team facilitates these discussions and ensures the scientific and practical issues about the CWEP is described.

Teams have successfully supported local BUILD program implementation of two rounds of the Higher Education Research Institute administered The Freshman Survey (2015, 2016), the Your First College Year survey (2016), Faculty Survey, and a tailored survey for juniors and seniors composed of critical items from these surveys designed to provide baseline data for students who were already enrolled at institutions when the BUILD program was initiated. The first follow-up survey of NRMN participants (late Fall 2016) has also launched. Refinement and “streamlining” of data collection efforts continues to minimize combined burden to participants of CWEP and local evaluation needs. This includes sites using data collected for the CWEP within local evaluation and the elimination of data elements from the CWEP not critical for measurement of Hallmarks or career progression.

The DPC has encountered multiple challenges as the CWEP has been implemented. Because the CEC will receive identifiable data to allow longitudinal evaluation (but only generate de-identified outcomes) the CEC, BUILD and NRMN all required Institutional Review Board (IRB) review and approval and the CEC worked closely with the Department of Education Family Policy Compliance Office to ensure the CWEP and other DPC activities were compliant with the Family Educational Rights and Privacy Act, commonly referred to as FERPA. Staff resources to support the CWEP were uniformly substantially lower than what is required for the ultimate scope of work. While the CEC assumes the primary responsibility for data collection, significant assistance and effort is required from staff at each BUILD program and NRMN to ensure rigorous and uniform data collection, which provides the foundation of longitudinal follow-up that may be as long as 20 years beyond the initial survey of individuals.

### Support of local evaluation

Each BUILD/NRMN awardee is conducting an independent site-level evaluation. During the first year of the DPC, CEC evaluation specialists reviewed each of the BUILD and NRMN site-level evaluation plans to ensure a consistent level of standardization and rigor across programs. Sites submitted completed detailed evaluation plans: program goals (at both the student and faculty levels), specific and measurable objectives, evaluation design, methods, key independent and dependent variables (process and outcome) and proposed measures and analysis, qualitative data collection plans, study limitations, and plans for use of study findings.

CEC and NIH reviewers rated each component of the detailed evaluation plan as acceptable, needing minor revisions, or needing major revisions. Reviewers and local evaluators then worked collaboratively over the subsequent 6 months to refine the evaluation plan within the context of the proposed program. In addition, the framework developed during the Consortium’s first year (e.g., Hallmarks, Arc of Success, and common measures) informed modifications to the local evaluation plan, such as inclusion of some psychosocial measures deemed interesting but less critical to the local evaluation. During the review process, efforts were made to align both site-level and consortium-wide evaluation efforts.

## Consortium coordination

The Consortium brings a range of resources, approaches, and expertise and will produce evaluation and pedagogical tools, scientific findings, and novel approaches to structure and process for BUILD/NRMN interventions. To leverage the resources and maximize dissemination of products, the CEC creates collaborative environments for the Consortium, supports the shared governance of the Consortium, and manages communication within the Consortium and more broadly to various communities such as other undergraduate institutions, professional societies, and funding agencies.

The CEC formed by pairing established leaders of health-related training programs with national experts in higher education research on faculty, undergraduate student experiences and STEM pathways. Various authors have described the importance of gathering diverse talent across disciplines and expertise when approaching complex problems [25]. Campbell’s fish scale model of omniscience also describes how science as a broad concept advances when overlapping narrow disciplines combine evidence [26]. Similarly, team science has been proposed as an approach to bring content and methodological expertise together from multiple perspectives in order to study multi-faceted problems [27]. As the goals and activities of the project developed, the CEC expanded to include those specializing in team science, evaluation, qualitative and quantitative methods, robust data systems, and translational science. This blend provides a cross-disciplinary approach as well as representation across career level to effectively inform decisions, essentially creating a new network from existing resources and expertise at the University of California, Los Angeles.

A significant resource for the CEC is the Higher Education Research Institute at UCLA (HERI), a group focused on improving outcomes for college students in the United States through longitudinal research. HERI is home of the Cooperative Institutional Research Program, a 50-year study of American college students. HERI manages administration of the surveys of the program. Collaboration with HERI enables the CEC to leverage expertise in longitudinal surveys with college students, infrastructure for such administration, a reporting structure, and an extensive bank of survey items with robust psychometric properties.

In addition to the expertise at HERI, the Director of the UCLA Computing and Technology Resource Laboratory is a CEC co-investigator. This group provides advanced technology for data collection and management as well as document sharing and storage platforms important for CEC coordination across the Consortium. The NIH-funded UCLA Clinical Translation Science Institute serves as a resource for the evaluation of clinical science training programs. CEC investigators and staff also work with this group, allowing shared resource and expertise. Finally, one of the senior investigators is principal investigator for the NIH-funded Resources Centers for Minority Aging Research Coordinating Center, which organizes and implements an Annual Investigators Meeting and provides the framework for reporting of uniform data and RCMAR Scholar productivity and achievements for junior faculty across six national sites, several of which have been active for 10–15 years. The CEC benefits from these experiences with uniform reporting and the tracking of scholars during key career transitions.

### Consortium governance

The DPC Executive Steering Committee is the decision-making authority for the DPC, providing general oversight and guidance to the DPC as a whole. There is representation of each BUILD program and of NRMN, CEC, and NIH on the Executive Steering Committee and it reviews and provides final approval on consortium-wide policies. Key Executive Steering Committee activities that are coordinated by the CEC are outlined in more detail below.

As described above, identifiable data is needed to chart the long-term progress of participants in DPC activities as well as from comparison groups. The Executive Steering Committee determined that adoption of a DPC Data Sharing Policy was important to provide clear communication among DPC entities regarding protections and expectations regarding data across the Consortium. The Data Sharing Policy establishes data collection, tracking and storage coordination requirements; delineates specific administrative, technical and physical safeguards to assure data security and confidentiality; describes access to and transfer of data to the CEC for use in the DPC’s evaluation; and provides a framework for use of DPC data, and delineates data ownership, rights, and grantee responsibilities. It includes detailed information on: i) period of the policy, ii) governance, authorities, data rights and compliance, iii) definitions, iv) data submission to the CEC; v) data security and use, vi) the method of data sharing, and vii) the process to handle any dispute that might arise.

Building on the Data Sharing Policy, the ESC established a Publications and Presentations Sub-Committee to provide structure for the management, use, and dissemination of data and results stemming from consortium-wide activities. Critical to the process is the establishment of policies that recognize and account for academic and scientific freedom at the individual institutions. The CEC support includes facilitation of the meetings, management of a repository of all consortium-related published and presented material, and tracking of the process for use of data through proposal to publication.

### Consortium communication and working groups

In cooperation with the rest of the DPC, the CEC developed a communication structure and resource sharing infrastructure that continues to evolve and expand. Some elements are accessible by the public while other resources are for communication only between DPC members. Electronic portals include the Consortium’s website, newsletter, Intranet web space, and BUILD/NRMN activity Tracker data management system. These tools support document storage and sharing, listserve updates, data storage and reporting, and streamlined consortium-wide communications, including announcements on intervention innovations and program activity implementation from each site. These portals also support the variety of virtual meetings critical to Consortium interactions. For broader issues of science and policy, the CEC hosts a monthly webinar series for all DPC programs and their partners on topics such as data management approaches at local institutions, advances in psychometrics, preliminary evaluation findings, challenges in program implementation, and recent evidence from other groups regarding diversity efforts. Finally, an Annual DPC Meeting is hosted by the CEC to meet goals that are collaboratively developed by Executive Steering Committee members and highlight working group priorities for any given year.

The CEC facilitates the shared work of the DPC through Working Groups formed around tasks or topics. Through these forums, DPC partners can share insights, examine research questions across institutions, and develop suggestions for activities and policies for the Consortium as a whole. Below are descriptions of some of these groups and how they are contributing to the work of the Consortium-Wide Evaluation Plan and the DPC.

The Communications Working Group facilitates communications within the DPC and with the broader scientific community. This group has developed a DPC branding strategy to increase visibility and reflect the excellence of DPC training activities and to ensure that the public relations strategy across partners is parallel. This also involves maintaining up to date shareable tools and channels of communication relevant to DPC and stakeholders; some tools currently utilized are the public website, an intranet for DPC internal communication and archival, and social media presence.

The Implementation Working Group focuses on the ways in which both local and consortium-wide evaluations are done. This group reviews all aspects of the Consortium-Wide Evaluation Plan implementation, including proposed design modifications, guidance on identification of participants and activities, and selection of data collection tools. Methods of secure data transfer and processes for ensuring high data quality are also routinely discussed. Finally, the group provides a forum for sites as they address the common challenges that arise with data collection (e.g. student and faculty survey burden, timing with exams), and evaluation implementation, particular within cooperative agreement structures.

The Recruitment and Retention Working Group provides BUILD and NRMN sites with guidance and support for program recruitment and retention in alignment with NIH’s diversity policy. Through this group, BUILD and NRMN investigators and staff are able to share best practices and challenges in the recruitment and retention of participants for the activities. One particular area of attention has been the recruitment of students from underrepresented groups while complying with legal requirements regarding equal access and race-neutral strategies. Programs are also given the opportunity to present on effective strategies around novel curricula, social media and branding, student support services, and program designated spaces (lounges, halls, etc.).

Affinity groups are comprised of consortium members from programs with common elements (e.g. development of novel curricula, diversity training for students and/or faculty, research training and support for students and/or faculty, mentor training, etc.) and/or consortium members with shared interest (e.g. implicit bias, stereotype threat, impact of family support). Affinity Groups promote the sharing and use of best practices and dissemination of findings through meetings, webinars, and publications. Capitalizing on domain-specific expertise within the consortium to enhance development of Consortium capacities situates the initiative for greatest impact and deepest reach.

## Conclusion

In summary, a consortium provides an incredible opportunity for team learning, growth and ultimately the generation of a deep and lasting impact on important problems of achieving diversity in biomedical education and training. Vital to securing the long-term success of the consortium, the CEC consults with the Executive Steering Committee on how best to facilitate the leveraging of consortium capacities and sharing lessons learned. This has led to the creation of several consortium-based workgroups led by the CEC using synchronous (e.g., webinars, conference calls) and asynchronous distance interactions (e.g., webinar recordings, email). The primary avenue for structured in-person interactions is at the DPC Annual Meeting, attended by key personnel (e.g., Principal Investigators, Program Managers, Directors, Support Staff, Evaluators) from the consortium and serves as a way to share scientific progress, engage in professional learning, and make plans for programmatic and consortium success.

In addition, the CEC approach to capacity building is founded on years of successful experiences developing, leading, and facilitating networks and partnered activities for a diverse group of biomedical and health science related programs, institutions, community-based organizations, and foundations. A systems approach is blended with systematic inquiry for learning in an effort to more fully develop the DPC capacity [2, 28–34]. Thus, the CEC has worked to harness the vast amount of skills and expertise that lie within the Consortium to support, sustain, and publicize the work. Some examples of this are best practices in outreach and recruitment, and structuring authentic research experiences that merit further evaluation to establish its evidence base. Ultimately, the CEC is responsible for the collection and rigorous assessment of DPC activities to understand and disseminate the evidence for how to support the increasing number of dedicated faculty, institutions, and strategic networks committed to making sure every trainee has opportunity to succeed in the biomedical sciences and to enhance the collective scientific brain trust to accelerate advances that will improve the nation’s health.
